# The association between internal migration and pulmonary tuberculosis in China, 2005–2015: a spatial analysis

**DOI:** 10.1186/s40249-020-0621-x

**Published:** 2020-02-17

**Authors:** Wei-Bin Liao, Ke Ju, Ya-Min Gao, Jay Pan

**Affiliations:** 1 West China School of Public Health and West China Fourth Hospital, Sichuan University, No. 17, Section 3, Ren Min Nan Road, Chengdu, 610041 Sichuan China; 2 Medical College, Northwest Minzu University, Lanzhou, China; 3 West China Research Center for Rural Health Development, Sichuan University, Chengdu, China

**Keywords:** Internal migration, Pulmonary tuberculosis, Spatial analysis, China

## Abstract

**Background:**

Internal migration places individuals at high risk of contracting tuberculosis (TB). However, there is a scarcity of national-level spatial analyses regarding the association between TB and internal migration in China. In our research, we aimed to explore the spatial variation in cases of sputum smear-positive pulmonary TB (SS + PTB) in China; and the associations between SS + PTB, internal migration, socioeconomic factors, and demographic factors in the country between 2005 and 2015.

**Methods:**

Reported cases of SS + PTB were obtained from the national PTB surveillance system database; cases were obtained at the provincial level. Internal migration data were extracted from the national population sampling survey and the census. Spatial autocorrelations were explored using the global Moran’s statistic and local indicators of spatial association. The spatial temporal analysis was performed using Kulldorff’s scan statistic. Fixed effects regression was used to explore the association between SS + PTB and internal migration.

**Results:**

A total of 4 708 563 SS + PTB cases were reported in China between 2005 and 2015, of which 3 376 011 (71.7%) were male and 1 332 552 (28.3%) were female. There was a trend towards decreasing rates of SS + PTB notifications between 2005 and 2015. The result of global spatial autocorrelation indicated that there were significant spatial correlations between SS + PTB rate and internal migration each year (2005–2015). Spatial clustering of SS + PTB cases was mainly located in central and southern China and overlapped with the clusters of emigration. The proportions of emigrants and immigrants were significantly associated with SS + PTB. Per capita GDP and education level were negatively associated with SS + PTB. The internal migration flow maps indicated that migrants preferred neighboring provinces, with most migrating for work or business.

**Conclusions:**

This study found a significant spatial autocorrelation between SS + PTB and internal migration. Both emigration and immigration were statistically associated with SS + PTB, and the association with emigration was stronger than that for immigration. Further, we found that SS + PTB clusters overlapped with emigration clusters, and the internal migration flow maps suggested that migrants from SS + PTB clusters may influence the TB epidemic characteristics of neighboring provinces. These findings can help stakeholders to implement effective PTB control strategies for areas at high risk of PTB and those with high rates of internal migrants.

## Background

Over the past two decades, despite considerable effort directed towards tuberculosis (TB) prevention and control, China has continued to experience one of the most significant TB endemics worldwide, with an estimated annual incidence of 67 per 100 000 population in 2015 [1]. In China, among the class A and B infectious diseases, TB has one of the highest notification rates and is a leading cause of death and poverty [2, 3]. The prevalence of TB slowly decreased between 2000 and 2010 [4]. However, due to population growth, the overall number of TB cases continues to increase and there are differences in prevalence between the east, central, and western regions of China [5].

With global economic development, migration flows have become increasingly intense; these play an important role in TB epidemiology, especially in China. With the reform of the household register system (*hukou* system), the number of internal migrants within China is growing rapidly [6]. In 2015, there were an estimated 247 million internal migrants in China (18% of the total population), and the growth trends are likely to continue [7]. Most internal migrants in China have traveled from rural areas to prosperous economic regions to pursue higher incomes and better lifestyle opportunities [8, 9]. Compared with permanent residents, internal migrants face substantial problems in accessing medical care, insurance, and social security [10]. According to previous studies, migrants are more vulnerable due to low income, poor working and living conditions, less education, and poor awareness of health protection [11–13]. Therefore, internal migration poses a challenge for TB control and prevention in China.

Spatial and spatiotemporal analysis of TB notification cases could provide crucial epidemiological information to guide interventions. In recent years, the geographical information system (GIS) and spatial statistics were used to detect the spatial characteristics of TB in China [14–18]. Several studies have demonstrated that TB is not randomly distributed. Furthermore, significant differences in cluster patterns were found between local residents and migrants, indicating that migrants may have been infected before their arrival [19, 20]. However, few studies have simultaneously focused on the spatial and spatiotemporal cluster patterns of TB, and the association between TB and internal migration in China.

This study aims to investigate trends in spatial clusters of sputum smear-positive pulmonary TB (SS + PTB) incidence between 2005 and 2015. Additionally, this study aims to identify the spatial distribution of internal migration flows and to evaluate the relationship between SS + PTB and internal migration at the provincial level. We hope that this study can contribute to informing effective medical resource allocation, guide the design of internal migration interventions, and identify factors underlying the spread of SS + PTB in high-risk areas.

## Methodology

### Data collection

Provincial incidence of notified SS + PTB cases was obtained from the national, web-based Notifiable Infectious Diseases Reporting Information System (NIDRIS), which includes sputum smear-positive, sputum smear-negative, sputum not done, and sputum culture-positive PTB data. Due to its high risk of transmission among the population, SS + PTB is of greatest concern; therefore, we focused on SS + PTB cases from 2005 to 2015 within China. Hong Kong, Macau, and Taiwan of China were not included in our analysis because of data accessibility. The classification of Eastern areas, Central areas and Western areas is based on the standard of the National Statistics Bureau.

The internal migration data were extracted from the national population sampling surveys in 2005 and 2015 (these surveys were conducted nation-wide and covered 16.99 million people (1.325% of the total population) and 21.31 million people (1.55% of the total population), respectively) and from the census in 2010. In the current study, internal migration was defined as a move from one province to another province within China. Internal migration was divided into emigration and immigration. We then calculated the proportion of emigrants (POE) and immigrants (POI) in the total population for each province. Other variables included gross domestic product (GDP) per capita (RMB 10 000; PCGDP), the proportion of people in the population with a college degree or higher (EDU), human immunodeficiency virus (HIV) incidence rate, urbanization rate (UR), and population density (persons per square kilometers; PD). Detailed information for these variables is shown in Tables 1 and 2.

**Table 1:** Specification of the variables

Variable	Definition of variable	Data source	Period
SS + PTB	Notification rate of SS + PTB	National Notifiable Infectious Diseases Reporting Information System	2005–2015
HIV	Human Immunodeficiency Virus incidence rate		
POE	The proportion of emigrant in total population for each province	national population sampling survey and census	2005, 2010, 2015
POI	The proportion of immigrant in total population for each province		
PCGDP	GDP per capita (RMB 10 000)	China Statistical Yearbook	2005–2015
EDU	The proportion of people with a college degree or above in total population		
UR	Urbanization rate		
PD	Population density (persons per square kilometers)		

*TB* Tuberculosis, *SS + PTB* Sputum smear-positive pulmonary TB, *POE* Proportion of emigrants, *POI* Proportion of immigrants, *PCGDP* Gross domestic product per capita, *EDU* College degree or higher, *HIV* Human immunodeficiency virus, *UR* Urbanization rate, *PD* Population density

**Table 2:** Descriptive statistics of independent variables

Variable	Mean	Standard deviation	Min	Max
Proportion of emigrants (%)	5.20	3.56	1.03	14.01
Proportion of immigrants (%)	6.67	9.06	0.3	39.53
GDP per capita(10 000 RMB)	3.42	2.33	0.51	10.8
Incidence of HIV (per 100 000)	3.8	4.53	0.04	18.95
Urbanization level (%)	51.03	15	22.67	89.3
Education level (%)	10.21	6.4	0.89	42.34
Population density (per square kilometer)	446.17	760.61	2.3	5663.19

*GDP* Gross domestic product, *HIV* Human immunodeficiency virus, *RMB* Renminbi

### Spatial autocorrelation analysis

Global Moran’s *I* is a measure of spatial autocorrelation developed by Moran [21]. It is widely used in public health to investigate spatial clusters of infectious diseases [22, 23]. We calculated global Moran’s *I* statistics in GeoDa (version 1.6.7, GeoDa Center for Geospatial Analysis and Computation, Arizona State University, AZ, USA) in order to examine the spatial autocorrelations of SS + PTB, POE, and POI in the study area. The equation for Moran’s *I* statistic is:

1 $$ I=\frac{n}{S_0}\frac{\sum_{i=1}^n{\sum}_{j=1}^n{w}_{ij}\left({x}_i-\overline{x}\right)\left({x}_j-\overline{x}\right)}{\sum_{i=1}^n{\left({x}_i-\overline{x}\right)}^2} $$

where *n* is the number of spatial units, *x*_*i*_ or *x*_*j*_ is the incidence of SS + PTB and the proportion of emigration or immigration in province *i* or *j*, *w*_*ij*_ is a matrix of spatial weight between province *i* and *j*, and *S*_0_ is the sum of all ${w}_{ij}:{S}_0={\sum}_{i=1}^n{\sum}_{j=1}^n{w}_{ij}$. The value of Moran’s *I* usually ranges from − 1 to 1, with positive values indicating a positive association and negative values indicating a negative association. A value approaching − 1 or 1 indicates a stronger association. The *Z*-statistic is used to test the significance of Moran’s *I*: *Z* = *I* − *E*[*I*]/*STD*[*I*], where *E*[*I*] =  − 1/(*n* − 1), *STD* = *E*[*I*^2^] − *E*[*I*]^2^.

The spatial relationships among the provinces were characterized by the spatial weight matrix. In our study, a first-order queen continuity weights matrix, which defines neighbors as those with either a shared border or vertex, was used for spatial weights. A queen weights matrix was constructed in GeoDa using the province level polygon-shaped file.

Global Moran’s *I* reveals the overall relationship of all the research units in the area. Local Moran’s *I* is a method to explore the local spatial distribution characteristics [24]. In this study, local Moran’s *I* was used to describe the local spatial autocorrelation; it is an indication of the extent of significant spatial clustering of similar values. The sum of local Moran’s *I* is proportional to global Moran’s *I*. The following equation was used to calculate the local Moran’s *I* statistic:

2 $$ I=\frac{\left({x}_i-\overline{x}\right)}{\frac{1}{n}\sum \limits_{i=1}^n{\left({x}_i-\overline{x}\right)}^2}\sum \limits_{j=1}^n{w}_{ij}\left({x}_j-\overline{x}\right) $$

where *n* is the number of spatial units, *x*_*i*_ or *x*_*j*_ is the proportion of emigrants or immigrants in province *i or j*, and *w*_*ij*_ is a matrix of spatial weight between province *i* and *j*. Like global Moran’s *I*, the value of local Moran’s *I* also ranges from -1 to 1, with a positive value indicating clustering of similar values and a negative value indicating the opposite. In this study, the local Moran’s *I* was used to make the LISA clusters maps. The cluster maps were presented in ArcGIS (version 10.2, ESRI Inc., Redlands, CA, USA).

### Analysis of spatial variation in temporal trends

The spatial variation in temporal trends was based on Kulldorff’s scan statistics, which are used for the identification of areas with exceptionally different temporal trends [25]. This method assumes that the average annual percentage change of SS + PTB within the scanning window is the same as that outside the window. A circular window is imposed on each location in turn; then, a number of circular windows that are flexible in both size and location are constructed. For each window, a likelihood is calculated, and the most likely cluster is defined as the window with the maximum likelihood, that is, the cluster least likely to be due to chance. Under the null hypothesis, the *P*-value is obtained from Monte Carlo hypothesis testing. In this study, the Poisson probability model was used, in which the number of cases in each location is under a Poisson distribution. The maximum number of replications for the Monte Carlo simulation was set to 999, and *P* <  0.05 was considered to be statistically significant [26].

It is reported that only 12% of immune-sensitized individuals actually develop clinical symptoms of TB, and the total number of PTB notification cases in China was approximately 11 million between 2005 and 2015 [27]. Therefore, this suggests that 100 million (8% of the total population in China) people in this time may have been sensitized and were at risk of developing TB. Many studies utilizing spatial temporal mapping suggest that the main guideline for selecting an optimal scanning window is reducing the overlapping areas, or that a single cluster should make up no more than 15% of the whole study area [28, 29]. Moreover, previous research in China at the prefecture level found that 11% was the optimal parameter for spatial cluster sizes, so we analyzed the notification rate of SS + PTB setting the maximum sizes from 8 to 11% of the total population at risk by increments of 1% [18]. When the maximum size is set at 8 to 11%, there are fewer overlaps, and the biggest cluster covered no more than 15% of all the provinces. As such, we choose 8% as the maximum spatial cluster size. The spatial variation in temporal trends of SS + PTB was examined using SaTScan (version 9.4.2, Kulldorff and Information Management Services, Inc., Boston, USA).

### Panel data analysis

A fixed-effects model was used to estimate the effects of internal migration, demographic factors, and socio-economic factors on SS + PTB incidence. The internal migration factors included emigrants and immigrants. Emigrant refers to those people who left their household registration place for more than half a year. Immigrant refers to those people who were settled in the current resident area for more than half a year.

We used GDP per capita (RMB 10 000), population density, education level, and urbanization level to reflect the social-economic situation. GDP represents the level of economic development of a region. Education and urbanization level can indirectly affect SS + PTB incidence via the effects of income or health education on TB prevention [30, 31]. The natural logarithm of each variable was used in the construction of the model. The basic descriptive statistics for these variables are presented in Table 2. Due to internal migration data accessibility, only three years (2005, 2010, and 2015) were included in the model. The model can be expressed as:

3 $$ {Y}_{i,t}=\alpha +{X}_{i,t}\beta +{\varepsilon}_{i,t} $$

where *Y*_*i*, *t*_ is the incidence of SS-PTB, *i* and *t* are the province and year, respectively, *α* is the intercept term, *X*_*i*, *t*_ is a vector of independent variables, *β* is the coefficients of the independent variables, and *ε*_*i*, *t*_ is the error term. The descriptive analysis and the fixed effects model were performed in Stata (version 12.0; StataCorp, TX, USA).

## Results

### Descriptive analysis of SS + PTB cases

A total of 4 708 563 SS + PTB cases were reported in China between 2005 and 2015, of which 3 376 011 (71.7%) were male and 1 332 552 (28.3%) were female. The number of male cases was twice that of female cases. The notification rates of SS + PTB decreased from 41.90 cases per 100 000 population in 2005 to 17.93 cases per 100 000 population in 2015, with an annual average rate of 29.84 per 100 000 population. In addition, a significant proportion of the SS + PTB infections were aged > 60 years old (around 30%) and 45 to 60 years old (around 26%). Among the reported cases, around two-thirds were peasants; the percentage of SS + PTB cases that were classified as a householder or unemployed increased over the years of the study (see Table 3).

**Table 3:** The demographic characteristics of SS + PTB cases in China from 2005 to 2015

	2005	2006	2007	2008	2009	2010	2011	2012	2013	2014	2015
Gender											
Male	384 688 (70.63)	313 534 (70.78)	308 354 (71.54)	315 401 (71.89)	344 360 (71.69)	328 155 (71.86)	289 079 (72.30)	500 574 (72.23)	221 248 (72.09)	194 846 (72.14)	175 772 (71.94)
Female	160 001 (29.37)	129 416 (29.22)	122 640 (28.46)	123 313 (28.11)	136 016 (28.31)	128 522 (28.14)	110 744 (27.70)	192 412 (27.77)	85 662 (27.91)	75 255 (27.86)	68 571 (28.06)
Age											
0–15 year	3850 (0.71)	2374 (0.54)	1915 (0.44)	1675 (0.38)	2045 (0.43)	1986 (0.43)	1604 (0.47)	1309 (0.38)	1059 (0.35)	933 (0.35)	893 (0.37)
15–30 year	123 338 (22.64)	108 044 (24.39)	102 315 (23.74)	104 391 (23.79)	109 913 (22.88)	105 017 (23.00)	92 617 (27.25)	74 868 (21.61)	65 097 (21.21)	54 807 (20.29)	47 009 (19.24)
30–45 year	139 311 (25.58)	112 978 (25.51)	105 667 (24.52)	104 351 (23.79)	110 715 (23.05)	103 156 (22.59)	87 885 (25.86)	72 674 (20.97)	62 546 (20.38)	52 179 (19.32)	44 312 (18.14)
45–60 year	129 020 (23.69)	101 314 (22.87)	101 757 (23.61)	106 326 (24.24)	120 887 (25.17)	116 542 (25.52)	104 238 (30.67)	91 130 (26.30)	81 904 (26.69)	73 211 (27.11)	66 616 (27.26)
> 60 year	149 170 (27.39)	118 240 (26.69)	119 340 (27.69)	121 971 (27.80)	136 816 (28.48)	129 976 (28.46)	113 479 (33.39)	106 512 (30.74)	96 304 (31.38)	88 971 (32.94)	85 513 (35.00)
Occupation											
Peasant	380 941 (69.94)	302 698 (68.34)	293 401 (68.08)	298 951 (68.14)	325 036 (67.66)	306 056 (67.02)	268 045 (67.04)	230 940 (66.65)	203 042 (66.16)	179 052 (66.29)	158 472 (64.86)
Worker	29 245 (5.37)	25 370 (5.73)	24 669 (5.72)	25 540 (5.82)	28 556 (5.94)	28 013 (6.13)	21 020 (5.26)	16 859 (4.87)	13 959 (4.55)	10 363 (3.84)	9616 (3.94)
Householder unemployed	27 154 (4.98)	23 876 (5.39)	24 300 (5.64)	25 972 (5.92)	29 809 (6.21)	30 352 (6.65)	30 734 (7.69)	29 249 (8.44)	32 112 (10.46)	34 001 (12.59)	32 508 (13.30)
Student	24 877 (4.57)	20 413 (4.61)	18 080 (4.19)	17 274 (3.94)	17 698 (3.68)	14 804 (3.24)	11 561 (2.89)	9066 (2.62)	7673 (2.50)	6627 (2.45)	5861 (2.40)
Migrant worker	16 887 (3.1)	16 018 (3.62)	17 080 (3.96)	17 197 (3.92)	18 312 (3.81)	18 872 (4.13)	12 206 (3.05)	8912 (2.57)	6209 (2.02)	3844 (1.42)	3447 (1.41)
Retiree	15 066 (2.77)	12 575 (2.84)	12 865 (2.98)	12 730 (2.90)	14 858 (3.09)	14 717 (3.22)	13 707 (3.43)	13 039 (3.76)	13 483 (4.39)	13 560 (5.02)	13 811 (5.65)
Others	50 519 (9.27)	42 000 (9.48)	40 599 (9.42)	41 050 (9.36)	46 107 (9.60)	43 863 (9.60)	42 550 (10.4)	38 428 (11.09)	30 432 (9.92)	22 654 (8.39)	20 628 (8.44)

*SS + PTB* Sputum smear-positive pulmonary TB

**Fig. 1:**
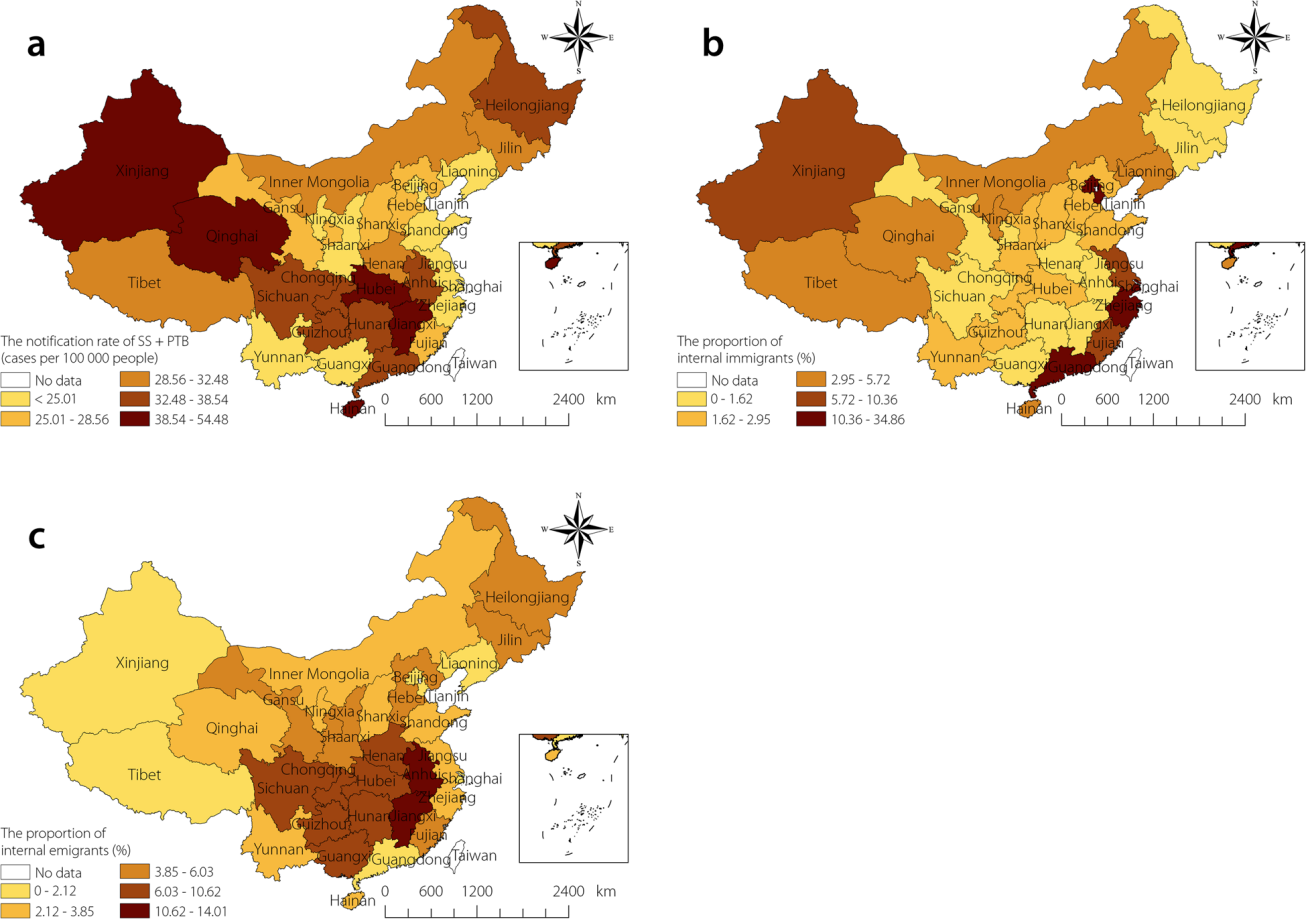
The annual average notification rate of SS + PTB and the proportion of emigrants/immigrants at province level in China, 2005–2015. **a** illustrates the notification rate of SS + PTB. **b** and **c** illustrate the proportion of immigrants and emigrants, respectively. TB: Tuberculosis; SS + PTB: Sputum smear-positive pulmonary TB

Figure 1 shows the spatial distribution of the annual average notification rate of SS + PTB, and the proportions of internal emigrants and immigrants in China at the provincial level from 2005 to 2015. There were obvious spatial variations in the annual average notification rate of SS + PTB, with rates ranging from 9.87 to 54.48 per 100 000 population. The highest SS + PTB notification rates were found in the provinces of Xinjiang, Qinghai, Hubei, Jiangxi, and Hainan, primarily in the northwest, southeast, and south of China.

Anhui (14.01%), Jiangxi (11.69%) provinces and Chongqing (10.62%) Municipality had the highest levels of internal emigrants. Further, provinces in the Central South had higher levels of emigrants (around 9.25% for each province). Provinces with the highest levels of internal immigrants were located in the eastern regions, such as in Beijing (31.15%), Tianjin (19.6%), Shanghai (34.86%), Zhejiang (18.51%), and Guangdong (20.19%) provinces. Provinces with lower levels of immigrants were also located in southeast areas close to Guangdong or Zhejiang. Interestingly, those provinces also had lower levels of internal emigrants.

### Global and local spatial autocorrelation

The global Moran’s *I* statistics showed positive spatial autocorrelations in SS + PTB in China each year (as presented in Table 4). Further, there was an increasing trend in global Moran’s *I*, which can be divided into three periods: 2005–2007, 2008–2009, and 2010–2015. The highest spatial autocorrelations were observed in 2011–2015, ranging from 0.319 to 0.388. Furthermore, the proportion of internal emigrants and immigrants also exhibited significant positive spatial autocorrelations each year (see Table 5).

**Table 4:** Globe Moran’s *I* statistics of SS + PTB in China, 2005–2015

Year	Moran’s *I*	*Z*-score	*P*-value
2005	0.169	1.8647	< 0.05
2006	0.162	1.7098	< 0.05
2007	0.199	1.8638	< 0.05
2008	0.143	1.4661	< 0.1
2009	0.233	2.3021	< 0.05
2010	0.243	2.3196	< 0.05
2011	0.319	3.1137	< 0.05
2012	0.335	3.1257	< 0.05
2013	0.388	3.6656	< 0.05
2014	0.387	3.5191	< 0.05
2015	0.384	3.6449	< 0.05

*SS + PTB* Sputum smear-positive pulmonary tuberculosis

**Table 5:** Globe Moran’s *I* statistics of emigrant and immigrant in China, 2005, 2010 and 2015

Year	Variable	Moran’s *I*	*Z*-score	*P*-value
2005	emigrant	0.300	2.9534	< 0.05
2005	immigrant	0.267	2.7822	< 0.05
2010	emigrant	0.281	2.6835	< 0.01
2010	immigrant	0.326	3.3589	< 0.01
2015	emigrant	0.288	2.7005	< 0.05
2015	immigrant	0.333	3.5324	< 0.01

**Fig. 2:**
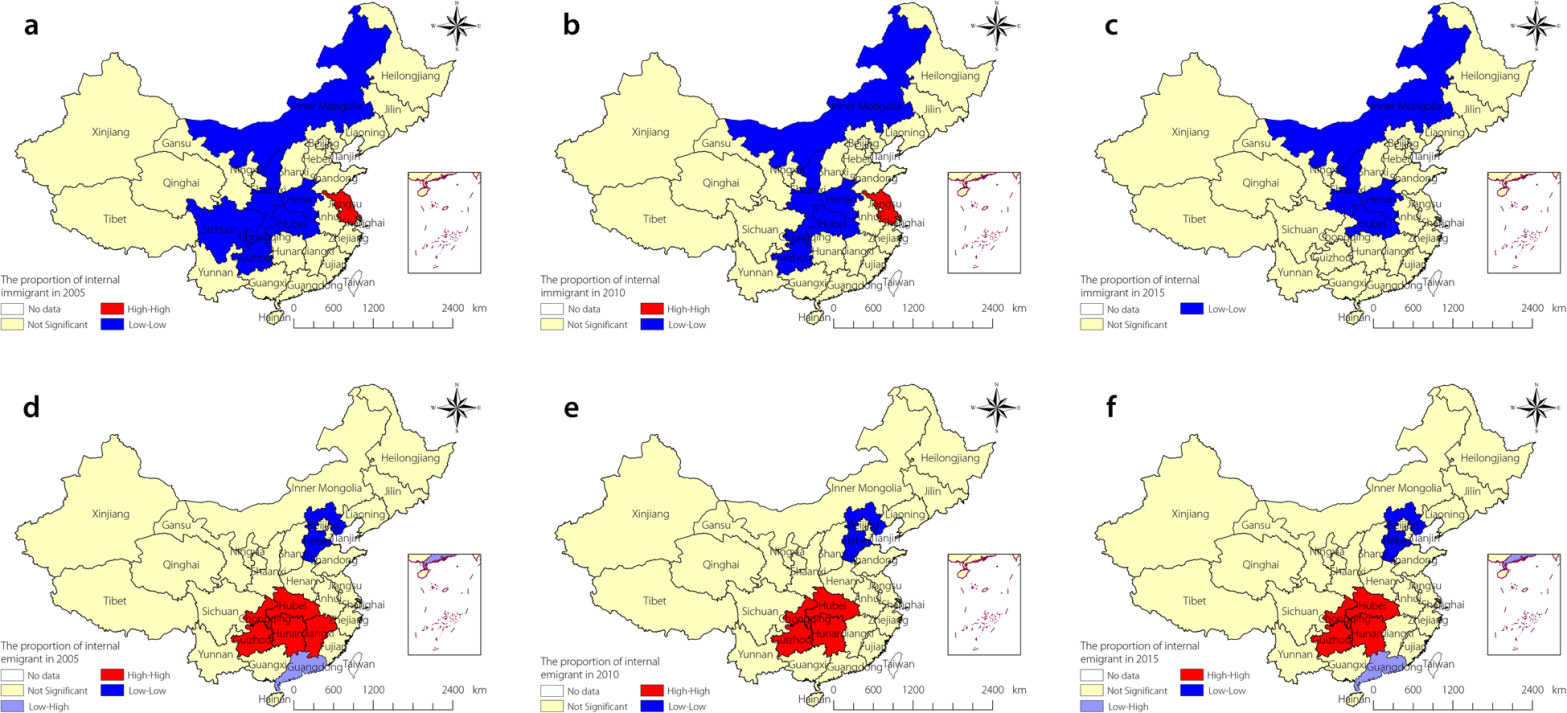
The LISA cluster map of the internal emigrant and immigrant in China. **a**, **b** and **c** for immigrant, **d**, **e** and **f** for emigrant. **a**, **b** and **c** show the spatial clustering of immigrants in 2005, 2010 and 2015, respectively. **d**, **e** and **f** show the spatial clustering of emigrants in 2005, 2010 and 2015, respectively. LISA: Local indicator of spatial association

Figure 2 shows the local Moran’s *I* statistics. We observed the stability of spatial clusters each year during the study period, and the clusters were stable within most provinces. Provinces such as Shaanxi, Henan, Chongqing, Guizhou, Inner Mongolia, and Hubei (mostly located in central China) showed a low-low type of relationship, indicating that these provinces had a low proportion of internal immigrants and that the surrounding provinces also had low proportions of immigrants. Jiangsu Province, which is located on the southeast coast of China, had a high-high type of relationship, meaning that a high proportion of immigrants were found in Jiangsu and that the surrounding provinces also had high proportions of immigrants. Chongqing, Guizhou, Hunan, and Hubei exhibited high-high types of relationships for the proportion of internal emigrants. On the other hand, Hebei Province exhibited a low-low relationship and Guangdong a low-high relationship.

### Spatial variation in temporal trends

**Fig. 3:**
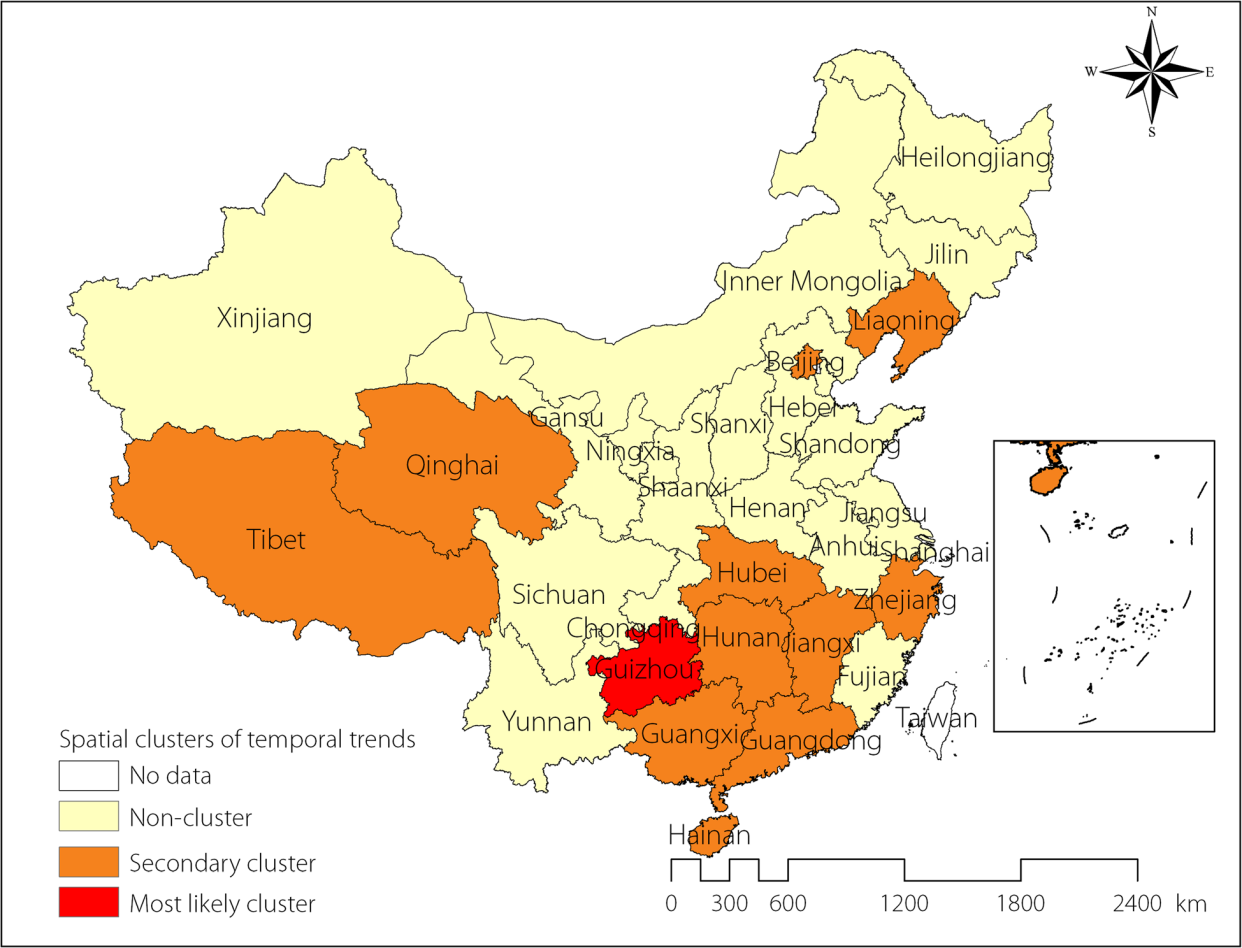
The spatial variation in temporal trends of smear positive PTB in China, 2005–2015. PTB: Pulmonary TB

From 2005 to 2015, there was a 6.96% annual average decrease in the notification rate of SS + PTB. We identified one most likely cluster and ten secondary clusters; two provinces/municipalities showed increasing annual trends; and nine provinces showed a slower decreasing annual trend compared to the outside time trend (Table 6). Guizhou and Beijing showed increasing annual average trends of 0.207 and 0.222%, respectively. Guangdong, Hunan, Jiangxi, Zhejiang, Liaoning, Qinghai, Hainan, Guangxi, Xizang, and Hubei showed decreasing annual average trends of 3.098, 3.256, 2.734, 3.581, 4.286, 4.082, 6.037, 4.358, and 6.483%, respectively. Figure 3 showed the spatial distribution of the most likely and secondary clusters. Most clusters were located in central and southern provinces of China; although Qinghai and Tibet are in west China, while Beijing and Liaoning are in northeast China.

**Table 6:** Spatial clusters of temporal trends of smear positive PTB in China, 2005–2015

Cluster	Province	Observed cases	Expected cases	Inside time trend	Outside time trend	RR	LLR	*P*-value
Most likely cluster	Guizhou	139 710	120 111.50	+ 0.207%	-7.186%	1.17	3910.53	< 0.001
Secondary cluster 1	Guangdong	370 418	327 992.83	-3.098%	-7.335%	1.14	3365.99	< 0.001
Secondary cluster 2	Hunan	275 328	214 349.02	-3.256%	-7.212%	1.30	2229.74	< 0.001
Secondary cluster 3	Jiangxi	202 934	146 151.46	-2.734%	-7.166%	1.41	2081.28	< 0.001
Secondary cluster 4	Zhejiang	136 317	172 774.33	−3.581%	-7.065%	0.78	883.59	< 0.001
Secondary cluster 5	Beijing	23 521	60 261.71	+ 0.222%	-6.977%	0.39	649.49	< 0.001
Secondary cluster 6	Liaoning	110 409	142 926.77	-4.286%	-7.034%	0.77	448.46	< 0.001
Secondary cluster 7	Qinghai	24 998	18 502.14	-4.082%	-6.980%	1.35	115.59	< 0.001
Secondary cluster 8	Hainan, Guangxi	168 914	184 250.00	-6.037%	-7.002%	0.91	84.00	< 0.001
Secondary cluster 9	Xizang	10 501	9717.49	-4.358%	-6.970%	1.08	39.88	< 0.001
Secondary cluster 10	Hubei	268 451	189 597.05	-6.483%	-6.986%	1.44	35.81	< 0.001

‘+’ means annual increase trend, ‘-’ means annual decrease trend

*PTB* Pulmonary tuberculosis, *RR* Relative risk, *LLR* Log-likelihood ratio

### The association between internal migration and SS + PTB

Three fixed-effect models were examined: one with the proportion of internal emigrants (model 1), one with the proportion of internal immigrants (model 2), and another with both the proportion of internal emigrants and the proportion of internal immigrants (model 3); panel regression results are presented in Table 7. The results showed that the proportion of emigrants and immigrants, per capita GDP, and the urbanization rate were found to be significantly associated with the SS + PTB rate. From model 1 and model 2, the coefficients for emigrants and immigrants were 0.632 and 0.536, respectively. Furthermore, we found that the proportion of emigrants was significantly positively related to SS + PTB, while the proportion of immigrants was not significantly related to SS + PTB in model 3. Further, model 1 had the highest R-square value; the model with only emigrants was able to explain 40% of the variation in SS + PTB rate.

**Table 7:** The result of fixed effect model

Variable	Model1	Model2	Model3
lnPOE	0.632 (0.165)^a^	Omit	0.412 (0.223)^c^
lnPOI	Omit	0.536 (0.171)^b^	0.355 (0.221)
lnPCGDP	−0.491 (0.275)^c^	−0.435 (0.227)^c^	−0.541 (0.260)^b^
lnHIV	1.516 (2.994)	0.107 (3.199)	0.437 (3.158)
lnUR	−1.253 (0.844)	−1.178 (0.729)	−1.304 (0.788)
lnEDU	−0.391 (0.175)^b^	− 0.492 (0.165)^b^	− 0.463 (0.169)^b^
lnPD	0.117 (0.087)	0.135 (0.114)	0.137 (0.090)
Intercept	1.419 (12.669)	7.602 (12.959)	6.059 (13.089)
No.Obs	93	93	93
R-squared	0.4	0.246	0.388

Robust stand-errors are in parentheses

*POE* Proportion of internal emigrants (%), *POI* Proportion of internal immigrants (%), *PCGDP* Per capita GDP (RMB 10000), *UR* Urbanization rate (%), *EDU* Proportion of population with college degree or above (%), *PD* Population density (1/km^2^), *HIV* Human immunodeficiency virus

^a^, ^b^ and ^c^ indicate the significance at 1%, 5%, and 10% level, respectively

### Internal migration flow maps and reasons

Based on the results of SS + PTB spatial cluster, the most likely cluster and the five secondary clusters were chosen to visualize the internal migration flow maps. Among these clusters, Guangdong, Beijing and Zhejiang are developed and prosperous provinces, and Guizhou, Hubei and Hunan are located in southern China, near Guangdong and Zhejiang provinces with a large immigrant population. The proportion of emigrants in these two provinces was significantly higher than the proportion of immigrants in Guizhou (POE: 10.22% vs POI: 2.08%), Hubei (POE: 9.01% vs POI: 1.94%), and Hunan (POE: 10.15% vs POI: 1.13%). In contrast, the proportion of immigrants was obviously higher than the proportion of emigrants in Beijing (POI: 31.15% vs POE: 1.66%), Zhejiang (POI: 18.51% vs POE: 3.45%), and Guangdong (POI: 20.19% vs POE: 1.03%).

**Fig. 4:**
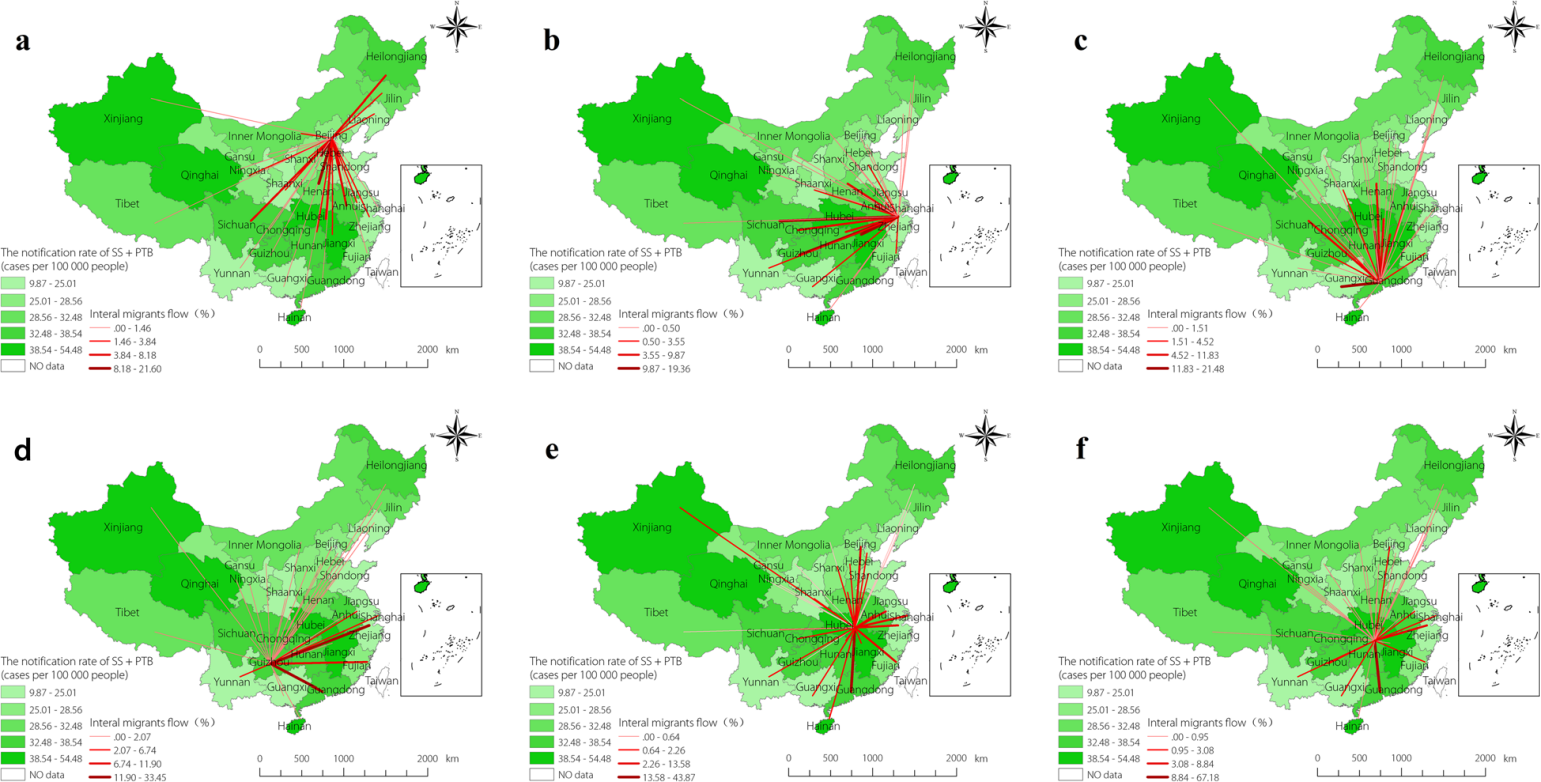
The internal migration flow of Beijing, Zhejiang, Guangdong, Guizhou, Hubei and Hunan province. **a**, **b** and **c** for immigrant flow, **d**, **e** and **f** for emigrant flow. **a**, **b** and **c** present the immigrant flow of Beijing, Zhejiang, Guangdong over 2005–2015, respectively. **d**, **e** and **f** present the emigrant of Guizhou, Hubei and Hunan over 2005–2015, respectively. TB: Tuberculosis; SS + PTB: Sputum smear-positive pulmonary TB

Figure 4 shows the flow of internal migrants for the six spatial clusters. The highest proportion of immigrants from Hebei (21.60%) flowed into Beijing, with immigrants from other spatial clusters accounting for 17.07%. Similarly, the highest proportion of immigrants from Anhui (19.36%) flowed into Zhejiang, and other spatial clusters accounted for 41.08%. The highest portion of immigrants from Hunan (21.48%) flowed into Guangdong, with immigrants from other spatial clusters accounting for 42.96%. In contrast, 33.45 and 26.56% of the emigrants in Guizhou flowed into Zhejiang and Guangdong, respectively. Further, 43.87 and 13.58% of the emigrants in Hubei flowed into Guangdong and Zhejiang, respectively. We also found that the highest proportion of emigrants from Hunan flowed into Guangdong (67.18%) and Zhejiang (8.84%).

Based on these results, it appears that internal migrants are more likely to flow into neighboring provinces or southern provinces. In Table 8, over 70% of internal migrants were leaving their place of household registration for work and business. Other reasons, such as study and training, only accounted for around 30% of internal migration.

**Table 8:** The reasons of internal migration in 2005, 2010 and 2015

Reasons	2005 (%)	2010 (%)	2015 (%)
Working and doing business	73.36	74.6	71.18
Study and training	1.44	4.40	8.64
Family members change residence following transferring of the workers	9.99	9.29	12.06
Marriage	4.00	2.56	2.65
House moving	0.54	0.86	0.81
A household registered in other’s family member	0.36	0.14	0.09
Other	10.31	8.08	4.57

## Discussion

In the current study, we identified spatial clusters and spatial variations in temporal trends in the distribution of SS + PTB cases in China between 2005 and 2015. There was a decreasing trend in the notification rate of SS + PTB; averaging an annual decrease of 6.96% in notifications. We also observed spatial variations in the distribution of internal migration. Further, these clusters were stable across the year in each year examined: 2005, 2010, and 2015. Compared with the flow of immigration, the flow of emigration was more consistent with the distribution of SS + PTB.

The global Moran’s statistic results indicated that although there was a decreasing trend in the SS + PTB notification rate, the distribution of the SS + PTB notification rate became increasingly clustered over time. One potential reason is the flow of patients from prefecture cities or counties to the provincial capital for better diagnosis and treatment; this could impact on the clustering of SS + PTB [8]. It should be noted that due to the time dimension limitation, local Moran’s test was not used to identify clusters of SS + PTB [32, 33]. Instead, we used the spatial variation of temporal trend method to evaluate the space-time distribution of SS + PTB based on Kulldorff’s scan statistical methodology.

The results showed that the most likely cluster and the ten secondary clusters were located in South, Northeast, and West China. Among these clusters, Guizhou, Hubei, Hunan, Guangxi, and Jiangxi provinces are in central-southern China. The main reasons for the high SS + PTB risk in these five provinces include low levels of socioeconomic development, the large proportion of the population living in poverty, and poor medical care [34]. Guangdong, Hainan, and Zhejiang are developed and prosperous provinces; internal migrants in these provinces accounted for a large part of the population, especially in Guangzhou and Hangzhou, the capital cities of Guangdong and Zhejiang, respectively. Research in Zhejiang has indicated that nearly one-third of reported TB cases are migrants [35]. Beijing was another cluster that exhibited an increasing trend in SS + PTB cases. It is believed that internal migration plays an important role in promoting growth in the TB epidemic in Beijing [6, 20]. Similarly, in Liaoning Province, the number of migrant TB cases increased by 30.55% since 2006, with the majority of cases observed in Dalian and Shenyang [36]. Further, we identified two clusters in Qinghai and Tibet, in the Northwest of China. Qinghai and Tibet are the largest political subdivisions in China, accounting for one-fifth of China’s total territory. Poor traffic conditions, uneven allocation of public health resources, and limited knowledge of TB are possible reasons for the high rates of TB in Qinghai and Tibet [32, 37]. With respect to traffic conditions, the Qinghai-Tibet railway was not operational until 2006; and the disparity of traffic infrastructure in such regions likely kept people from seeking medical help.

We also found significant positive spatial autocorrelations in both the proportion of internal emigrants and immigrants, and the clusters where this was noted were concentrated in central-southern China. In contrast, some high-high clusters for the proportion of emigrants overlapped with the low-low clusters for the proportion of immigrants. This may suggest that people in those areas are more likely emigrate to other provinces, or that these areas are less attractive for internal immigrants.

We observed that the proportions of emigrants and immigrants were statistically significant in the models 1, 2 and 3, while emigrants were more significant than immigrants. Previous studies have demonstrated that the number of rural-to-urban migrants has been increasing steadily, and this has a significant impact on urban TB epidemics [6, 19, 20]. In this study, internal migration was assessed in terms of emigration and immigration. The results of the fixed effects model suggested that the model with the emigration variable was able to explain more variation of SS + PTB. Further, we compared the spatial temporal clusters of SS + PTB and high-high clusters of emigration. Some emigration clusters overlapped with the SS + PTB clusters, primarily in central-southern China. Therefore, we found that the migrant population in those clusters was at an increased risk of SS + PTB infection and transmission.

The results of the emigration flow maps indicate that migrants from central-southern clusters would likely prefer to emigrate to Guangdong and Zhejiang. On the contrary, the results of the immigration flow maps indicate that migrants in Guangdong, Zhejiang, and Beijing primarily come from neighboring provinces. These results are consistent with the provincial distribution of TB cases in the migrant population in China [38]. Therefore, strategies to control TB in these areas must consider the characteristics of internal migration flow. Since the reform of the Chinese economy, southeast coastal provinces have become the most economically developed areas in China. When people are aware that local health services cannot meet their needs, they will seek better health services in different provinces. By the end of 2015, it was estimated that there were around 8.22, 32.01, and 23 million internal migrants in Beijing, Guangdong, and Zhejiang, respectively. As shown in Table 8, the main reasons for internal migration were the pursuit of work and business. The high-speed China railway also makes it convenient for people to migrate to neighboring provinces within a few hours.

Per capita GDP and education level were found to be statistically significant in the models 1, 2 and 3, indicating that economic development, and improved awareness of TB could help to alleviate the SS + PTB epidemic. Our results are consistent with other studies [15, 39, 40]. Evidence indicates that TB is a poverty-related disease, with an average treatment period of 6 months [41]. Due to different medical insurance systems, internal migrants who come from country areas are usually covered by the new rural cooperative medical system, and their health insurance is generally not transferable to a new location. Further, migrants are required to return to their hometowns (*houkou* registered places) to seek medical treatment under their insurance. However, due to low level medical and transportation costs, thus, they are less likely to go back to their hometown to benefit from health insurance [42]. Despite the available strategies for free diagnosis and treatment of TB, such as the Direct Observed Treatment Short Course (DOTS) strategy, there remains a lack of knowledge about these among the migrant population. This is a critical problem in China and is a barrier to accessing TB care [43, 44]. The national plan to control TB 2011–2015 required 85% of the public to possess some knowledge of TB by the end of 2015. However, national and local surveys show that poor awareness of TB remains in most areas.

Urbanization, the HIV incidence rate, and population density were predictors that not statistically significant in the models 1, 2 and 3. We found that HIV incidence rate was positively associated with SS + PTB. This may be due to a disparity in the distribution of SS + PTB and HIV; for example, there may be a high prevalence of TB/HIV co-infection in poor areas [45]. Due to data accessibility limitations, we did not have data on the PTB/HIV coinfection incidence rate. Population density was also positively correlated with SS + PTB. Similar results have been reported in other studies [46–48]. Due to poor financial conditions, migrants are more likely to be living in crowded places that favor the spread of *Mycobacterium tuberculosis*; this explains the association between population density and SS + PTB. In addition, we observed that urbanization was negatively associated with SS + PTB. This means that urban areas that have well-developed public health infrastructure, better-qualified health care workers, and where most of the residents are covered by medical insurance (such as urban resident basic health insurance or urban employee basic health insurance), are less at risk of SS + PTB [44, 49]. In contrast, the prevalence of TB in rural areas is consistently higher than in urban areas in China [4, 50, 51]. Due to their slower developing economies, these areas have limited healthcare resources and continuing government input is required to improve TB prevention and control efforts in these areas.

Several limitations of this research should be noted. First, this study did not include migrant SS + PTB cases, and this may affect the distribution of PTB. This is because the national health and family planning commission began to conduct dynamic surveys of migrants in 2009; thus reliable data on the migrant population was not available in 2006–2009. Second, the spatial level analysis was conducted by province, and the observed cluster patterns may depend on the spatial scale chosen. It is preferable to identify spatial distribution by smaller geographical units such as a county. Third, the selection of the maximum circle size of the scanning window may influence the results of the spatial-temporal scan statistics. In this study, the maximum size of the scanning window was set as 8%; further investigation is needed to test the sensitivity of the scanning window. Finally, this was an ecological study examining the association between SS + PTB rate and risk factors; the potential ecological fallacy is inevitable.

## Conclusions

In short, there was a decreasing trend in the notification rate of SS + PTB between 2005 and 2015. We found spatial-temporal clustering of SS + PTB and spatial variation in internal migration in China. The SS + PTB clusters were mainly located in central-southern China, and the internal migration clusters were mainly located in central inland China. The proportions of emigrants and immigrants were positively correlated with SS + PTB, while per capita GDP and education level were negatively correlated with SS + PTB. The proportion of emigrants was a more significant predictor of SS + PTB and could explain more variation in SS + PTB compared to the proportion of immigrants. Further, we found that the SS + PTB clusters overlapped with emigration clusters, and the internal migration flow maps suggested that migrants from SS + PTB clusters may influence the TB epidemic characteristics of neighboring provinces. Therefore, we recommend that policymakers acknowledge that migrants are a vulnerable population group. Cooperative efforts should be strengthened between provinces where there are high proportions of emigration and immigration in order to enable effective TB control. Further research is needed to explore the TB epidemic characteristics associated with internal migration based individual migrant data, particularly in central-southern China.

## Data Availability

The monthly reported all-forms PTB cases from January 2005 to December 2015 in each of 31 provinces of mainland China were obtained from the web-based national Notifiable Infectious Diseases Reporting Information System (NIDRIS). We would like to share statistical results of this study. If anyone needs these data, please contact the corresponding author for a soft copy.

## Abbreviations

EDU: College degree or higher
GDP: Gross domestic product
GIS: Geographical information system
HIV: Human immunodeficiency virus
LISA: Local indicator of spatial association
NIDRIS: Notifiable Infectious Diseases Reporting Information System
PCGDP: Gross domestic product per capita
PD: Population density
POE: Proportion of emigrants
POI: Proportion of immigrants
SS + PTB: Sputum smear-positive pulmonary TB
TB: Tuberculosis
UR: Urbanization rate
